# Immune microenvironment heterogeneity reveals distinct subtypes in neuroblastoma: insights into prognosis and therapeutic targets

**DOI:** 10.18632/aging.205246

**Published:** 2023-11-27

**Authors:** Yanlan Yang, Huamei Li, Donghui Zheng, Xuemei Li, Hongyan Liu

**Affiliations:** 1 Department of Hematology and Oncology, Shenzhen Children’s Hospital, Shenzhen, Guangdong, PR China; 2 Department of Hepatobiliary Surgery, The Affiliated Drum Tower Hospital, Medical School, Nanjing University, Nanjing 210008, PR China

**Keywords:** neuroblastoma, tumor microenvironment, subtypes, prognosis, drug response

## Abstract

Background: Neuroblastoma (NB) is a childhood cancer originating from immature nerve cells in the sympathetic nervous system. Current clinical and molecular subtyping methods for NB have limitations in providing accurate prognostic information and guiding treatment decisions.

Results: To overcome these challenges, we explored the microenvironment of NB based on the knowledge-based functional gene expression signatures (Fges), which revealed heterogeneous subtypes. Consensus clustering of Fges activity scores identified three subtypes (Cluster 1, Cluster 2, and Cluster 3) that demonstrated significant differences in prognosis compared to mainstream subtypes. We assessed the immune infiltration, immunogenicity, CD8T cytotoxicity, and tumor purity of these subtypes, uncovering their distinct biological functions. Cluster 1 and Cluster 2 exhibited higher immunoreactivity, while Cluster 3 displayed higher tumor purity and poor prognosis. Gene ontology annotation and pathway analysis identified immune activation in Cluster 1, epithelial-mesenchymal transition (EMT) in Cluster 2, and cell cycle processes in Cluster 3. Notably, the impact of EMT activity on prognosis may vary across NB subtypes. A classification model using XGBoost accurately predicted subtypes in independent NB cohorts, with significant prognostic differences. *GPR125*, *CDK4*, and *GREB1* emerged as potential therapeutic targets in Cluster 3. *CD4K* inhibitors showed subtype-specific responses, suggesting tailored treatment strategies. Single-cell analysis highlighted unfavorable clinical features in Cluster 3, including high-risk classification and reduced cytotoxicity. Suppressed interactions between monocytes, macrophages, and regulatory T cells were observed, affecting immune regulation and patient prognosis.

Conclusion: To summarize, we have identified a new independent prognostic factor in NB that underscores the significant correlation between tumor phenotype and immune contexture. These findings deepen our understanding of NB subtypes and immune cell interactions, paving the way for more effective treatment approaches.

## INTRODUCTION

Neuroblastoma (NB) is a childhood cancer that originates from the neural crest during embryonic development and primarily occurs in the sympathetic nervous system [1]. Common symptoms include the presence of an abdominal mass, discomfort, and swelling, along with ocular changes, bone pain, fever, fatigue, and irritability. Early diagnosis and treatment are crucial for improving the prognosis in children with this condition. Clinically, diagnosing NB involves both laboratory tests and imaging. Laboratory tests check for specific substances like Vanillylmandelic acid/Homovanillic acid (VMA/HVA) in urine, blood, and bone marrow. Imaging techniques include ultrasound, computed tomography (CT) scans, magnetic resonance imaging (MRI), metaiodobenzylguanidine (MIBG) scans, bone scans, and positron emission tomography (PET)-CT scans. These tools are crucial for accurately diagnosing NB, determining its stage, and planning the appropriate treatment. In terms of the affected population, NB is most commonly diagnosed in young children, with a slight male predominance, affecting individuals of all racial backgrounds. Notably, Black and Native American patients with NB tend to have a higher incidence of high-risk disease, leading to poorer Event-Free Survival (EFS) outcomes compared to white patients [2, 3]. In particular, due to the variability in clinical presentation and disease progression, 5-year survival rates for NB show considerable diversity. Research studies indicate that the 5-year survival rate for NB is below 50% [4, 5].

Considering the heterogeneous nature of NB, patient-specific staging to address different treatment strategies is clinically relevant. Currently, three primary approaches for staging and classifying NB are commonly employed: The International Neuroblastoma Staging System (INSS), the International Neuroblastoma Risk Group (INRG), and the Children’s Oncology Group (COG) risk stratification [6, 7]. The INSS classification categorizes tumors into four stages based on their size, depth, and aggressiveness to forecast patient prognosis and formulate treatment plans. INRG classifies patients into low, medium, and high-risk groups. Low-risk patients may not require immediate treatment, whereas medium-risk individuals often undergo a combination of chemotherapy, surgery, and radiation. High-risk cases, the most severe, demand intensive treatment, involving potent strong chemotherapy, surgery, radiation, immunotherapy, and stem cell transplants [8]. Conversely, COG risk stratification relies on the biological and molecular genetic traits of the tumor to devise treatment strategies and assess prognosis [6, 7, 9]. Additionally, there are supplementary molecular subtyping techniques applicable to NB, encompassing classifications related to chromosomal ploidy and groupings contingent on mutational conditions [10–13]. In the context of high-risk NB patients, a significant frequency of genetic variances is observed within specific chromosomal regions, specifically 1p, 11q, and 17q. Specifically, the presence of 11q deletion in NB cases without *MYCN* amplification, often indicates a highly malignant form with an unfavorable prognosis [14, 15]. Familial NB is associated with mutations in the *PHOX2B* and *ALK* genes, which can be inherited and are frequently accompanied by other syndromes related to neurocognitive development [16, 17]. High-risk NB patients often exhibit elevated levels of *BDNF* and *TrkB*, correlating with the malignancy and poor prognosis [18]. Alterations in the RAS and p53 pathway genes are commonly observed in high-risk and recurrent NB cases. *SETD8*, a histone methyltransferase, may be associated with a poor prognosis in *MYCN* non-amplified NB [19]. Additionally, the *BARD1* gene and its isoform protein, *BARD1β*, are linked to high-risk NB and could potentially serve as therapeutic targets [20]. *DDX1* and *DDX4* have also been identified in specific NB cases, exhibiting associations with distinct disease characteristics [21]. Nevertheless, further research is essential for gaining a comprehensive understanding of their functions and clinical significance. Furthermore, mutations and deletions in *ARID1A/B* have been correlated with drug resistance and elevated mortality rates in NB, indicating the potential pivotal roles of these genes in NB development [22]. Notably, HVA and VMA are breakdown products of dopamine and are useful for diagnosing NB. Elevated ferritin, lactate dehydrogenase (LDH), and neuron-specific enolase (NSE) levels are associated with NB, helping in diagnosis, assessing disease extent, and predicting prognosis. When considered alongside clinical and imaging findings, these biomarkers offer valuable insights into NB’s status and progression.

Although the acceptance of genomic analysis in clinical decision-making is on the rise, current classification systems and biomarkers also have limitations in accurately portraying the biological and molecular genetic characteristics of tumors, hindering the development of personalized treatment plans and precise prognostic assessments [23]. Therefore, the introduction of a new classification strategy for NB is highly anticipated. Recently, transcriptome analysis provides a deeper understanding of tumor complexity and heterogeneity, presenting an opportunity to discover new biomarkers for the development of innovative treatment strategies [24]. The tumor microenvironment (TME) plays a pivotal role in clinical outcomes and treatment response. Immune cells infiltrating the tumor can profoundly impact tumor progression and the success of cancer therapy by exerting both pro- and anti-cancer effects [25, 26]. Researches have been demonstrated that cancer-associated fibroblasts (CAFs) and vascular signaling of stromal cells can impact outcomes [24, 27]. Therefore, deciphering the tumor immune microenvironment profile can enhance individualized targeted and immunotherapy strategies. Notably, computational methodologies are particularly essential in oncology, enabling the discovery of novel diagnostic, prognostic, and treatment agents. These techniques leverage complex biological data and extensive datasets to identify molecular signatures associated with various cancer types, improving early and precise cancer diagnostics. Furthermore, they assist in modeling cancer progression, predicting patient outcomes, and refining personalized treatment approaches through data-driven analyses and machine learning [28–31]. At present, the investigation of NB subtypes through immune microenvironment transcriptomic features is ongoing, the integration of computational methodologies and transcriptome data plays a crucial role in understanding the heterogeneity of the NB microenvironment.

In our research, based on the knowledge-based functional gene expression signatures (Fges) provided by Bagaev et al. [24], which reflect the major functional components of TME, we systematically investigated the activity of these gene sets in NB patients and their association with prognosis based on the computational strategy. By utilizing the activity of Fges, we effectively identified NB patients into three distinct subtypes and explored the molecular features and functional heterogeneity in the microenvironment. To ensure generalization to other NB patient cohorts, we have developed a reliable classification model, and combined with drug sensitivity analysis, provides potential clinical guidance for treatment. Specifically, our study focused on utilizing single-cell expression profiles to uncover the key molecular mechanisms that contribute to the prognostic differences observed among NB subtypes.

## MATERIALS AND METHODS

### Data source and preprocessing

In the present study, we obtained transcriptome sequencing expression data and clinical data for NB patients from “Therapeutically Applicable Research to Generate Effective Treatments” (TARGET) database (named TARGET-NB cohort, *n* = 151), with expression data normalized by FPKM (Fragments Per Kilobase of exon model per Million mapped fragments). TARGET-NB cohort data were mainly used to explore TME and as the training data for investigating the potential subtypes of NB. Also, GSE49710 (*n* = 357) [32–34] and GSE85047 (*n* = 266) [35] cohorts from the Gene Expression Omnibus (GEO) accessed using “GEOquery” (version 2.60.0) [36], and clinical data corresponding to these two datasets were also acquired. Background correction and quantile normalization were performed using *rma* function from “affy” package (version 1.70.0) [37] for expression data, and combined with clinical data to evaluate the prognosis performance of derived novel NB subtypes. More detailed information can be found in Supplementary Table 1.

To investigate the intrinsic molecular mechanisms of heterogeneity in NB subtypes, we further obtained a single-cell dataset provided by Verhoeven et al. [5] from https://github.com/shenglinmei/NB.immune.atlas/. This dataset includes 46,134 cells from 17 NB patients and covers ten major immune cell types, including B cells, ILC3, Macrophages, mDC, Monocytes, NK cells, Plasma cells, Cytotoxic T cells (Tcyto), Helper T cells (Th), and Regulatory T (Treg) cells.

### Gene set activity score and signature score

We used the GSVA package (version 1.40.1) [38] with the “method = gsva” to examine the activity score of given gene sets for NB patients. The signature scores of a list of gene sets were evaluated using the *AddModuleScore* function from the Seurat package.

### Consensus clustering for investigating potential subtypes of NB

To investigate the potential molecular subtypes of NB, we obtained 29 cell types closely associated with the TME and their corresponding signature genes from https://github.com/BostonGene/MFP/blob/master/signatures/gene_signatures.gmt (Supplementary Table 2). We used the “ssgsea” method in the GSVA package to assess the activity of these cell types in NB patients, transforming the gene expression matrix into an activity score matrix for different cell types in the microenvironment. In this matrix, rows represent cell types, columns represent samples, and each entry represents an activity score. Based on this activity matrix, we utilized unsupervised consensus clustering, implemented by the “ConsensusClusterPlus” R package (version 1.62.0) [39] with parameters “clusterAlg = pam, distance = pearson, pItem = 0.8”, to derive intrinsic subtypes of NB in the TARGET-NB cohort. In the present study, we determined the optimal number of clusters by varying the number from 2 to 6 and selecting the most stable consensus matrices and unambiguous cluster assignments across permuted clustering runs.

### Kaplan-Meier survival curve

Kaplan-Meier (KM) survival analysis, along with the log-rank test, was used to determine if the subtypes showed a significantly different overall survival (OS). For gene expression data, the optimal cutoff point for group stratification was determined using the *surv_cutpoint* function in the “survminer” package (version 0.4.9) [40]. Statistical significance was determined by a *p*-value less than 0.05.

### Differentially expressed genes (DEGs) associated with the Fges-derived subtypes

To identify DEGs across subtypes, we utilized the *FindAllMarkers* function from “Seurat” package (version 4.3.0) [41]. Specifically, genes with adjusted *p*-values below 0.01 and an absolute log2FC greater than 1 were deemed significantly differentially expressed.

### Functional enrichment analysis

To examine the distinct biological processes and pathways that exhibited significant differential expression among subtypes, we employed the “clusterProfiler” package (version 4.0.5) [42] to convert gene symbols into Entrez ids using the *bitr* function. Subsequently, gene ontology (GO) and KEGG pathway enrichment analysis was conducted using the *enrichGO* function with the “ont = BP” parameter and *enrichKEGG* function, respectively. GO and KEGG terms that had adjusted *p*-values below 0.01 were regarded as significantly enriched.

### Estimation of tumor purity, immune score, immunophenoscore, and cytotoxicity score

The estimation of immune score, and tumor purity of NB patients was conducted by utilizing the “ESTIMATE” package (version 1.0.13) [43], as specified in the official manual. The Immunophenoscore (IPS) employs various markers of immune response or immune tolerance to measure the immune activity within an NB patient. A higher score of IPS indicates a greater level of immunogenicity [44]. The cytotoxicity score for each NB patient was determined by utilizing GSVA tool with *GZMA* and *PRF1* markers [45].

### Prediction of Cluster 1&2 and Cluster 3 subtypes for NB patients

Given that the Cluster 3 identified by the TARGET-NB cohort demonstrated the worst prognosis, we introduced the XGBoost model [30] to distinguish between Cluster 1&2 and Cluster 3 categories for individual NB patients. The following steps were taken: (1) The TARGET-NB cohort was randomly split into training and testing sets at a 3:1 ratio. We used the “FindAllMarkers” function from the Seurat package to identify genes that were significantly different in the Cluster 1&2 and Cluster 3 groups (adjusted *p*-value ≤ 0.01) as the features for the training set. (2) We applied the XGBoost model to the training set using 10-fold cross-validation, as implemented by the “xgb.cv” function from the “xgboost” R package (version 1.6.0.1). The parameters used were “nfold = 10, objective = multi:softprob, max.depth = 10, eval_metric = mlogloss” [46]. The model with the highest area under the curve (AUC) value was retained, and its performance was further evaluated using the testing set and two independent cohorts (GSE49710 [32–34] and GSE85047 [35]).

### Significance of novel NB subtypes in drug sensitivity

To assess the drug sensitivity of the Cluster 1&2 and Cluster 3, we utilized the “oncoPredict” package (version 0.2) [47] with the “batchCorrect = eb” parameter to determine the half-maximal inhibitory concentration (IC50) values of Palbociclib and Ribociclib anti-tumor drugs for the three cohorts of NB, respectively. This package employs a gene expression and drug sensitivity modeling algorithm for cell lines in the Cancer Genome Project, with the aim of predicting clinical chemotherapeutic response.

### Cell-cell interactions analysis

We employed CellChat [48] (version 1.6.1) to determine cell-cell interactions based on the expression of known ligand-receptor (L-R) pairs in different cell types. Briefly, the input for CellChat consisted of gene expression data of cells along with their assigned cell types. Initially, we identified overexpressed ligands or receptors within specific cell groups and projected the gene expression data onto a protein-protein interaction network. Overexpressed L-R interactions were identified when either the ligand or the receptor was overexpressed. Subsequently, CellChat facilitated the inference of biologically significant cell-cell communication by assigning a probability value to each interaction and conducting a permutation test. Finally, the resulting communication networks were visualized using a circle plot, and the signaling pathways were visualized using a bubble plot.

### Statistical analysis

The study utilized standard statistical tests such as Student’s *t*-test, Wilcoxon rank-sum test, log-rank test, and Cox proportional hazards regression to analyze both clinical and expression data. These analyses were carried out using R4.2.2.

### Availability of data and material

We collected gene expression data and corresponding clinical information for the NB cohorts from publicly available databases, as outlined in Supplementary Table 1. Especially, for this study, we utilized R 4.2.2 for the analysis, and the relevant R packages are described in detail in the Materials and Methods section. All codes are available upon request to the corresponding author.

## RESULTS

### Fges portray the heterogeneity of the TME in NB patients and the strong association with prognosis

**Figure 1:**
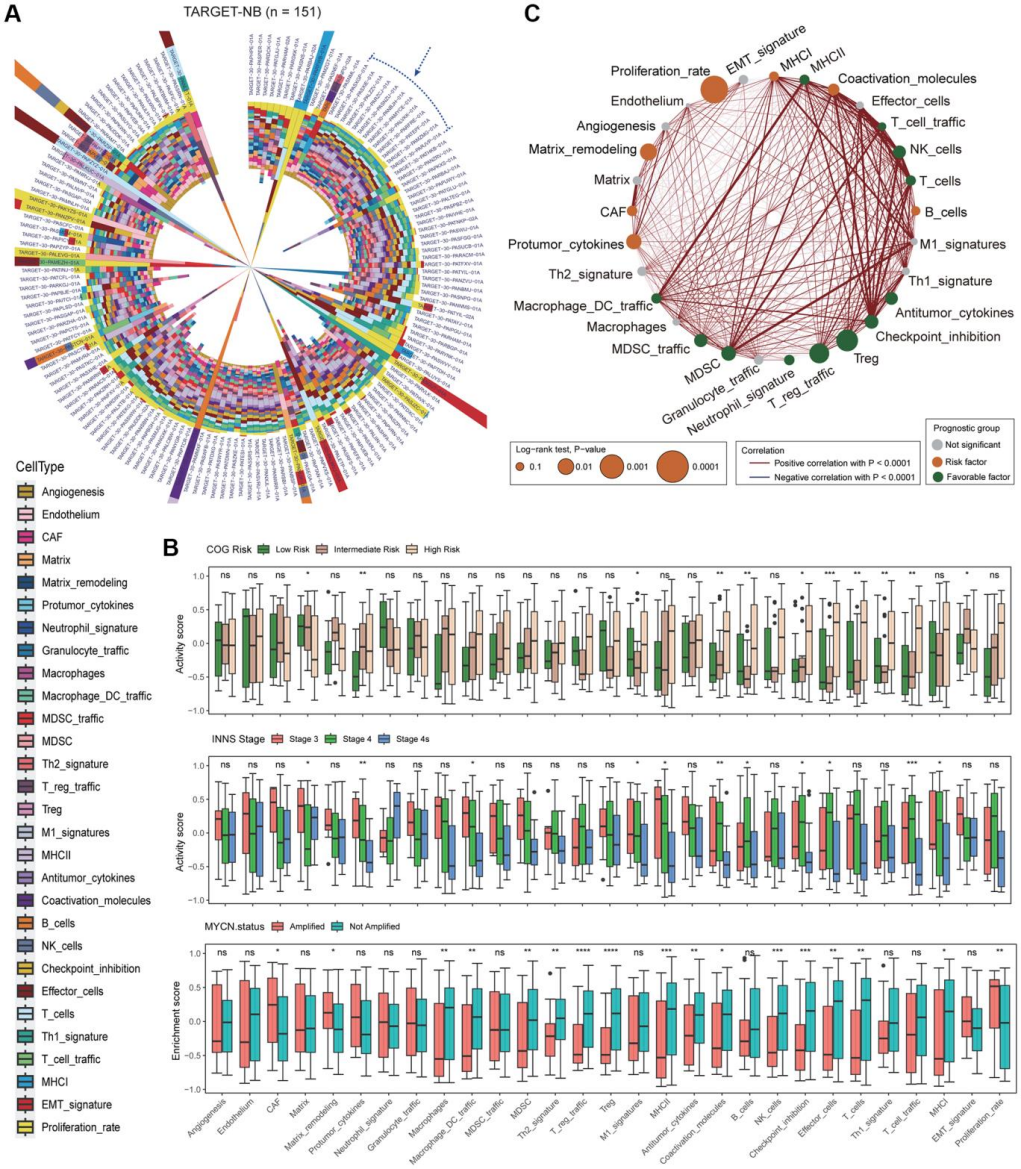
**Analysis of Fges activity scores reveals the heterogeneity of the neuroblastoma microenvironment and its significant impact on prognosis.** (**A**) Circular stacked bar plot shows the distribution of activity scores of 29 Fges in TARGET-NB patients. Different colors represent different gene sets (Supplementary Tables 2 and 3). (**B**) Box plot shows the distribution of activity scores of Fges in TARGET-NB patients in different groups, with *p*-values obtained by Wilcoxon rank-sum test. Abbreviation: ns: not significant; ^*^p < 0.05; ^**^p < 0.01; ^***^p < 0.001; ^****^p < 0.0001. (top panel) COG risk groups; (middle panel) INSS stages; (bottom panel) *MYCN* amplified or not. (**C**) Network plot displays the interaction of Fges within the microenvironment of NB and their influence on the prognosis of patients. Positive Pearson correlations between sets of genes are represented by red lines, while significant negative associations are denoted by blue lines. The significance of each gene set in predicting the prognosis of NB patients is depicted by the size of the corresponding circle. Gene sets that have a protective effect are shown in green, while those that pose a risk are represented in red; otherwise, they are shown in gray.

To investigate the TME of NB, we utilized a transcriptomic-based analytical platform and selected 29 functional gene sets (Fges) that represented the major functional components of the tumor, as well as immune, stromal, and other cell populations from previous study [24] (Supplementary Tables 1 and 2). We then employed GSVA (method = “ssgsea”) to assess the activity score of Fges in TARGET-NB patients, and found that the distribution of scores varied across patients, indicating the heterogeneous TME in NB patients (Figure 1A; Supplementary Table 3; see Materials and Methods). Interestingly, patients within the blue dashed box, showed a high degree of homogeneity in the distribution of activity scores, suggesting the presence of distinct subtypes in the microenvironment of NB (Figure 1A). We raised the question of whether the mainstream classifications could explain the heterogeneity in the microenvironment of NB patients. To investigate this, we analyzed the Fges score in COG risk categories, and found that, except for Protumor_cytokines, Coactivation_molecules, B_cells, Effector_cells, T_cells, Th1_signature, and T_cell_traffic, most (22/29) of Fges were no significant differences among low, intermediate, and high-risk categories under *p*-value ≤ 0.01. This suggests that COG risk stratification may not fully reflect the changes in the microenvironment of NB (Figure 1B). Also, we examined differences in Fges score across INSS stages and box plots showed that, similar to COG risk subtypes, most Fges (26/29) did not show significant differences. However, nearly half (13/29) of the Fges exhibited significant differences in *MYCN* status (amplified or not amplified), suggesting that the subtypes portrayed by *MYCN* may be more closely linked to the TME of NB (Figure 1B). Notably, we observed no Fges that shared significant differences in COG, INNS and *MYCN* (Figure 1B).

We also analyzed the interactions between Fges and their prognostic implications, showing that most Fges were synergistic in the NB microenvironment, such as MHC II and Macrophage_DC_traffic (Figure 1C; Supplementary Table 4; see Materials and Methods). The MHC II molecule is involved in promoting T-cell activation and clearance of tumor cells. In NB patients, higher levels of MHC II expression are associated with longer survival (Figure 1C). Similarly, increased expression of Macrophage_DC_traffic, a gene signature related to immune cell infiltration, is also a predictor of better prognosis in NB (Figure 1C). Typically, T_reg and T_reg_traffic hinder the immune response against tumors and their high levels are frequently linked to poor prognosis. However, our observations indicate that these two factors actually have a significant favorable impact on the prognosis of NB patients, suggesting the complexity and heterogeneity of NB patients. On the other hand, Proliferation_rate is a major risk factor, as a high proliferation rate suggests that the tumor has a greater growth capacity, faster growth rate, and higher malignancy level.

To sum up, the activity of Fges is responsive to the microenvironment heterogeneity in NB patients and is closely associated with prognosis. These findings have the potential to improve our understanding of NB tumor mechanisms and to guide the development of new classification strategies.

### Derivation of novel subtypes associated with distinct microenvironmental characteristics of NB from the perspective of Fges

**Figure 2:**
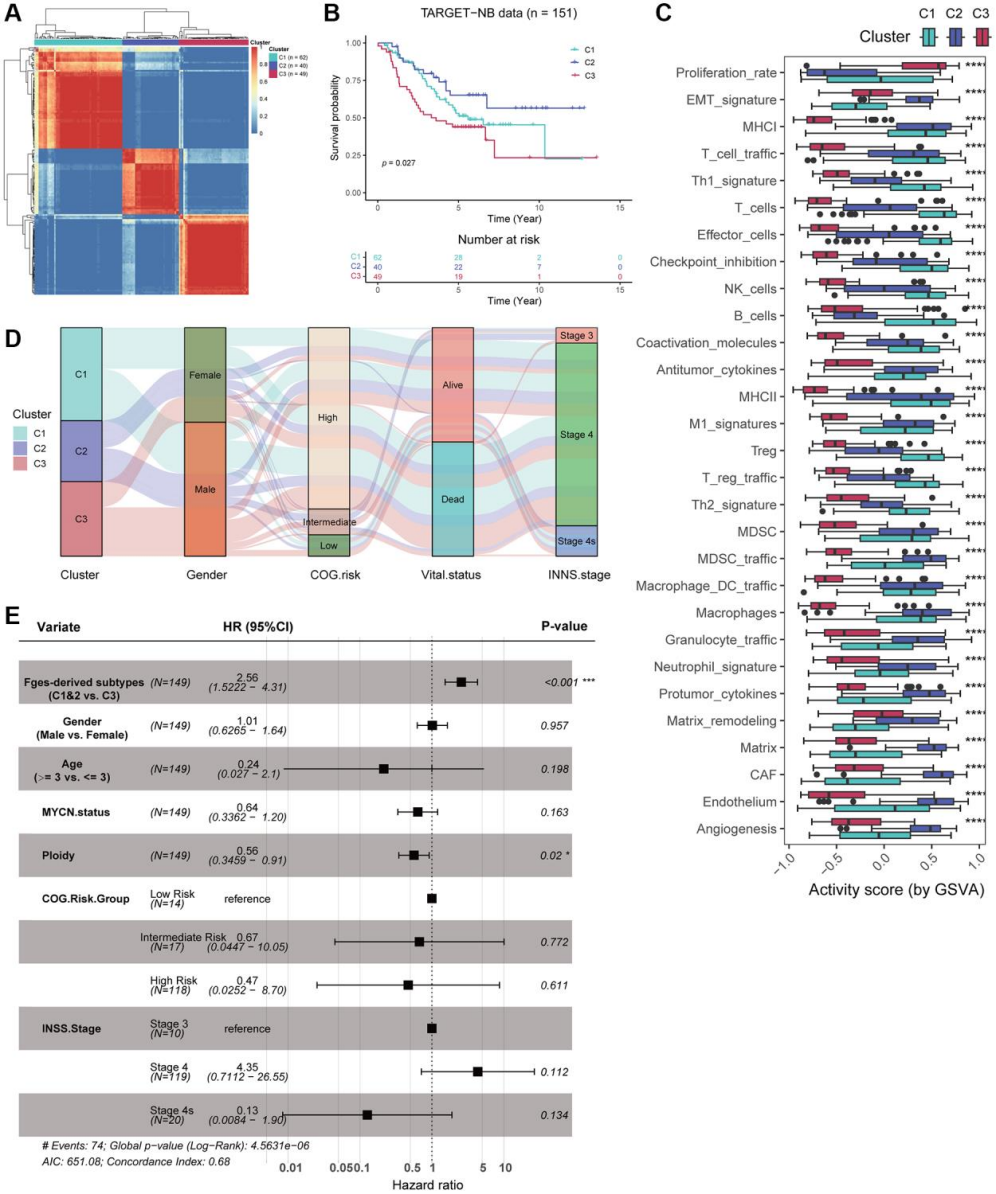
**Consensus clustering identified three novel subtypes based on Fges activity scores.** (**A**) Heatmap shows the consensus clustering matrix of the TARGET-NB cohort based on the activity score of Fges. (**B**) KM curve shows that the three Fges-derived subtypes exhibit significant differences in prognosis. *P*-value was obtained by log-rank test. (**C**) Box plot shows the distribution of activity scores of Fges in three Fges-derived subtypes. *P*-value was obtained by Wilcoxon rank-sum test. ^****^p < 0.0001. (**D**) Sankey plot shows the associations between the Fges-derived subtypes and clinical features, including gender, COG risk groups, survival status, and INSS stages. (**E**) Forest plot shows the independent effect of Fges-derived subtypes, along with other clinical features as well as classical groupings, on the prognosis of patients with neuroblastoma. This was determined through multivariate Cox regression analysis.

To investigate if functional gene sets (Fges) can reveal molecular subtypes of NB, distinct from those identified previously based on clinical and molecular features, we conducted a consensus clustering analysis on the activity scores of Fges, resulting in the categorization of patients into three subtypes: Cluster 1, Cluster 2, and Cluster 3 (Figure 2A; Supplementary Table 3). We first examined the sample sizes of the three new subtypes, with Cluster 1 having the highest number of samples (*n* = 61) and Cluster 2 the lowest (*n* = 40), with a more evenly distributed overall distribution (Figure 2A; Supplementary Table 5; see Materials and Methods). Moreover, significant differences in the prognosis of these three subtypes were observed, with Cluster 2 showing the best prognosis, followed by Cluster 1, and Cluster 3 having the worst (Figure 2B; see Materials and Methods). Furthermore, we analyzed the distribution of Fges activity scores in the three subtypes, and revealed significant differences in all functional gene sets. Interestingly, Cluster 3 showed the lowest overall activity scores, while EMT_signature, Matrix, and CAF showed higher activity scores in Cluster 2, suggesting that EMT may help to inhibit the growth and spread of NB tumors, leading to a better prognosis (Figure 2C). We also found that immune cells, such as T_cells and effector_cells exhibited high activity scores in Cluster 1, but their prognosis was slightly worse than that of Cluster 2, suggesting that high degree of immune infiltration in this subtype may indicate that the tumor or infection is suppressing the immune system, leading to a poorer prognosis for patients (Figure 2C).

By comparing the associations between the three subtypes and other clinical features and classical staging, including gender, COG, survival status, and INSS stages, we found that Cluster 3 was more likely to occur in males and had higher mortality rates (Figure 2D). However, we also noticed that Cluster 1, Cluster 2, and Cluster 3 did not differ significantly in COG risk groups as well as INSS stages, suggesting inherent differences between the subtyping identified by Fges and COG as well as INNS (Figure 2D). Moreover, to determine whether Fges-derived subtyping could be used as an independent prognostic risk factor in clinical studies of NB, we employed multiple Cox regression with covariates including Fges-derived subtypes, Gender, Age, *MYCN*, Ploidy, COG, and INSS (Figure 2E). The results indicated that Cluster 3 was a significant risk factor relative to Cluster 1 and Cluster 2, and there was no significant effect on prognosis except for Ploidy, suggesting that Fges-derived NB subtypes is an independent prognostic factor and has a good advantage in distinguishing prognosis significantly compared to other clinical characteristics and typing strategies (Figure 2E).

### Biological differences across Fges-derived NB subtypes

**Figure 3:**
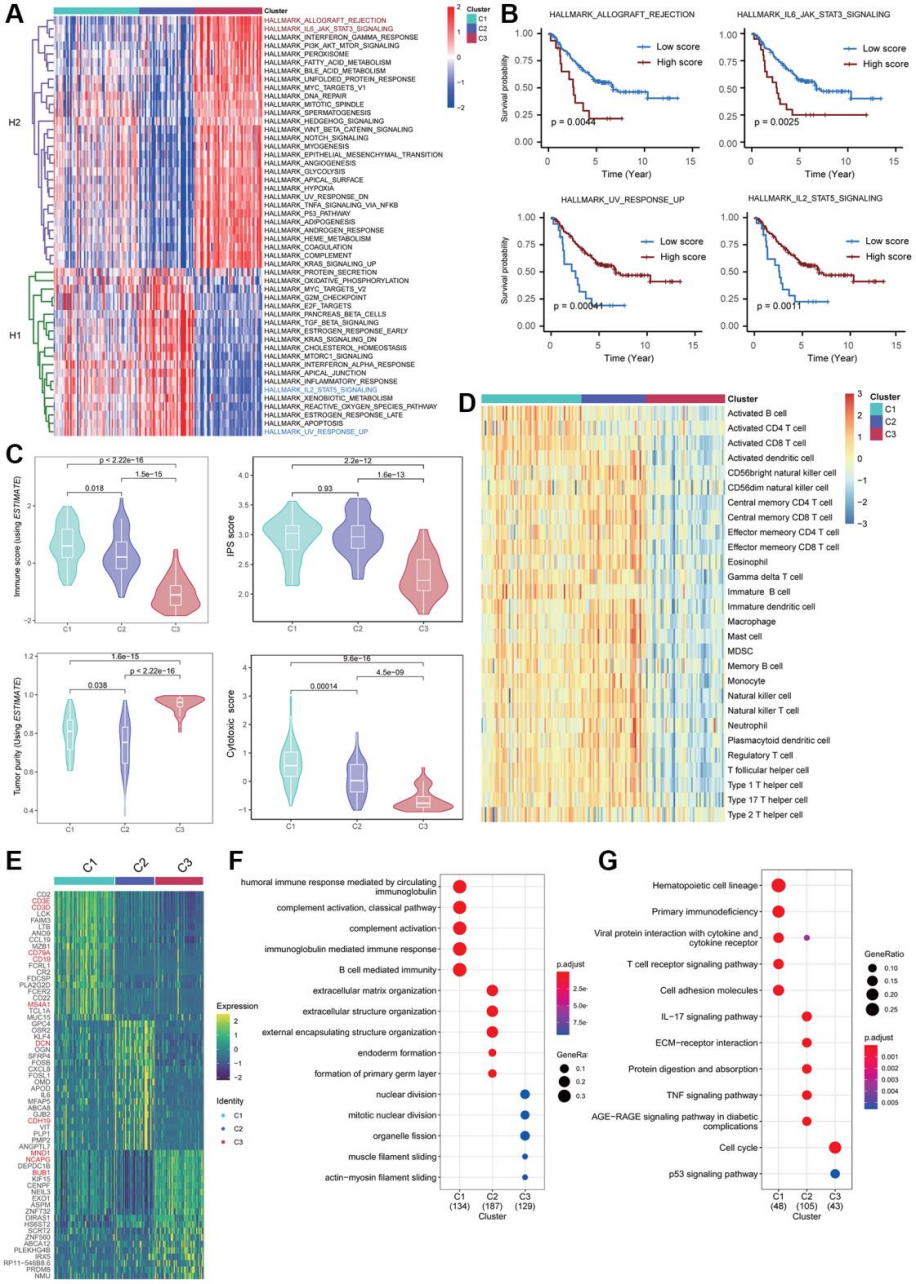
**Biological heterogeneity across Fges-derived subtypes.** (**A**) Heatmap displays the activity scores of cancer hallmark pathways for Cluster 1, Cluster 2, and Cluster 3 subtypes. These pathways were collected from MSigDB database, and can be classified into two categories, H1 and H2. (**B**) KM curves demonstrate the significant impact of high activity in the four cancer hallmark pathways on the prognosis of NB patients. The *p*-values were obtained through log-rank tests. (**C**) Violin plots show the distribution of different biological features in the three Fges-derived subtypes, including immune score, IPS, tumor purity, and cytotoxic score. *P*-values were obtained by a *t*-test. (**D**) Heatmap of activity scores of 28 immune cell gene sets provided by a previous study [44], among the Fges-derived subtypes. (**E**) Heatmap shows the expression of the top 20 highly expressed genes among three Fges-derived subtypes. (**F**) Bubble plot shows the biological processes significantly involved in the three Fges-derived subtypes of NB. (**G**) Bubble plot shows the pathways significantly involved in the three Fges-derived subtypes of NB.

Next, we investigated the activity score of cancer hallmark pathways collected from MSigDB in three Fges-derived subtypes using “ssGSEA” method, and hierarchical clustering clearly showed that these pathways could form two distinct categories (Figure 3A; see Materials and Methods). One category (i.e., H1) had a higher level of activity in Cluster 1 and Cluster 2, which are potential protective factors in NB patients, while the other category (i.e., H2) had a higher level of activity in Cluster 3, indicating risk factors (Figure 3A). To validate this finding, we selected two cancer hallmark pathways from each category, including HALLMARK_ALLOGRAFT_REJECTION, HALLMARK_IL6_JAK_STAT3_SIGNALING, HALLMARK_UV_RESPONSE_UP, and HALLMARK_IL2_STAT5_SIGNALING, and observed their KM curves of OS. The results showed that high activity in Cluster 3 for HALLMARK_ALLOGRAFT_REJECTION and HALLMARK_IL6_JAK_STAT3_SIGNALING exhibited a significantly worse prognosis, while high activity in HALLMARK_UV_RESPONSE_UP and a higher degree of activity in HALLMARK_IL2_STAT5_SIGNALING (Clusters 1&2) predicted a better prognosis, consistent with our previous inference (Figure 3B). Overall, these analyses suggest that hallmark pathways highly relevant to tumor progression exhibit distinct roles and patterns in Fges-derived subtypes.

Moreover, we delved deeper into the biological diversity among the three Fges-derived subtypes, encompassing immune infiltration, tumor purity, IPS, and cytotoxicity. The findings revealed that Clusters 1&2 displayed substantially higher (*t*-test, *p*-value ≤ 0.01) immune infiltration, immunogenicity and cytotoxicity scores, while Cluster 3 exhibited significantly lower immune infiltration and the greatest level of tumor purity (Figure 3C; see Materials and Methods). To reinforce these results, we also evaluated the activity of 28 immune cell types (782 marker genes) obtained from Charoentong P et al. [44] within the three subtypes and demonstrated that Clusters 1&2 had a greater degree of immunity in comparison to Cluster 3 (Figure 3D; see Materials and Methods). These analyses suggest that elevated immune infiltration is strongly linked to a favorable prognosis, indicating that NB is a “hot” tumor, consistent with prior investigations [49, 50].

To elucidate the biological functions and pathways involved in the three Fges-derived subtypes, we conducted differential gene expression analysis. This revealed that T cell and B cell markers such as *CD3D*, *CD3E*, *CD19*, and *CD79A* were highly expressed in Cluster 1, while fibroblast-related genes such as *DCN* and *CDH19* were highly expressed in Cluster 2 (Figure 3E; Supplementary Table 6). Additionally, genes associated with meiosis, including *MND1*, *NCAPG*, and *BUB1*, were significantly highly expressed in Cluster 3 (Figure 3E; Supplementary Table 6). Further functional enrichment analysis showed that Cluster 1 was associated with immune response processes and T-cell activation pathways, Cluster 2 was significantly associated with extracellular matrix organization and ECM-receptor interactions, and Cluster 3 was significantly associated with the cell cycle, indicated that the Fges-derived isoforms exhibit significant biological heterogeneity (Figure 3F, 3G; Supplementary Table 7; see Materials and Methods).

### Predicting Fges-derived subtypes with XGBoost model

**Figure 4:**
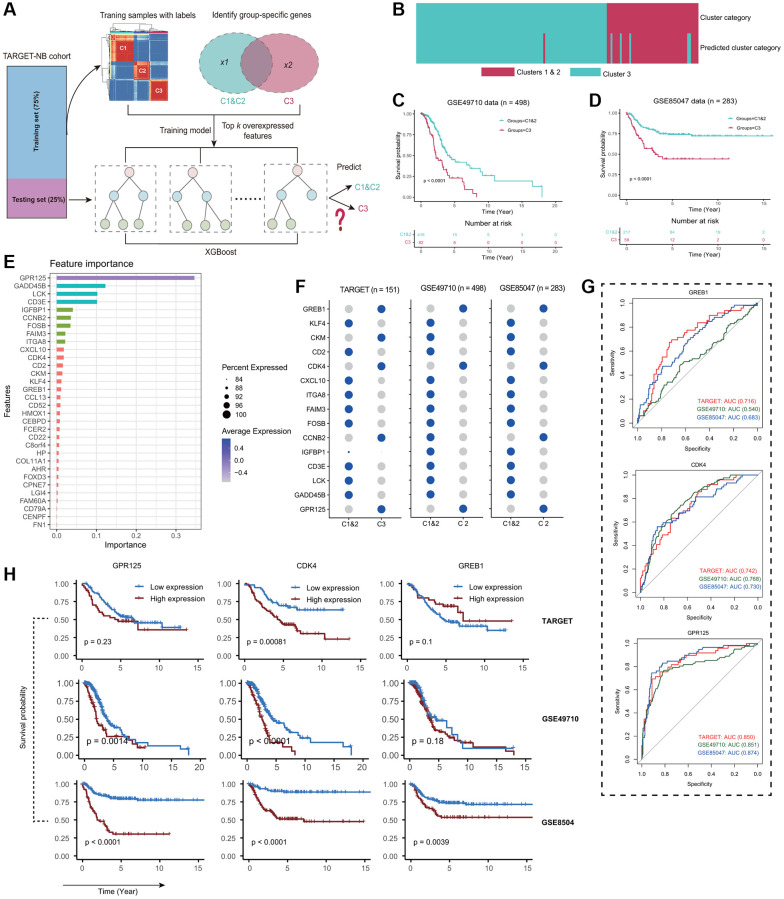
**Independent datasets validation of the reliability of Fges-derived subtypes.** (**A**) Schematic of the XGBoost model for predicting Fges-derived subtypes (see Materials and Methods). (**B**) Heatmap shows whether a particular sample, predicted by XGBoost classifier from the training set, falls under Cluster 3 (light blue) or Cluster 1&2 (purple), out of the 151 total samples. (**C**, **D**) The association between predicted clusters and survival was tested using Kaplan-Meier survival curves for predicted Cluster 3 versus Cluster 1&2. *P*-values were from log-rank tests. (**E**) Bar plot shows the importance ranking of the top 35 feature genes (ordered by Gain index) filtered by XGBoost in the training set. (**F**) Bubble plots show the expression of the top 15 important feature genes in the three NB cohorts. The depth of the color indicates the average level of expression of one gene in a particular subtype, and the size of the circle indicates the percentage of that gene expressed in a particular subtype. (**G**) ROC curves depict how accurately the expression of three genes can predict subtypes Cluster 1&2 in comparison to Cluster 3 based on the TARGET-NB cohort. (top) *GREB1*; (middle) *CDK4*; (bottom) *GPR125*. (**H**) KM curves show the prognostic impact of high and low expression of *GREB1*, *CDK4* and *GPR125* genes in three NB cohorts. (top panel) TARGET-NB; (middle panel) GSE49710; and (bottom panel) GSE85047.

Our objective was to develop a precise and sensitive method for predicting patient Fges-derived subtypes without relying on unsupervised clustering. To achieve this goal, we utilized the TARGET-NB cohort and trained our model using the XGBoost algorithm to predict Cluster 1&2 and Cluster 3 subtypes (Figure 4A; see Materials and Methods). Using differential gene expression analysis, we identified a set of genes that were able to predict whether a sample belonged to Cluster 3 with high sensitivity and specificity (Supplementary Table 8). We then integrated these genes into our XGBoost model for training. Our model achieved an accuracy of 0.953 (0.906–0.981) in predicting subtypes (Figure 4B; see Materials and Methods). We observed that 93.6% of samples assigned to Clusters 1&2 by clustering were predicted to be 1&2 by our model, while 85.7% of samples assigned to Cluster 3 through clustering were found in Cluster 3 (Figure 4B). By applying our model to two other NB testing cohorts GSE49710 and GSE85047, and combining it with clinical information, we were able to demonstrate significant differences in prognosis for Cluster 1&2 and Cluster 3 subtypes (Figure 4C, 4D). These results were consistent with our observations in the TARGET-NB cohort, which suggests that Fges-derived subtypes are highly consistent across NB cohorts. In summary, our XGBoost model refined Cluster 3 and provided a single-sample predictor that can be used for every patient in the clinic.

Next, we analyzed the importance of feature genes in our XGBoost prediction model and found that *GPR125* had the highest importance (Figure 4E). Previous studies have confirmed that this gene plays a crucial role in the development of the nervous system and is involved in the migration and differentiation of neural stem cells, indicating that *GPR125* is a vital biomarker for the clinical study of Cluster 3 [51–53]. Additionally, we observed the expression of these significant signature genes and found that *GREB1*, *CDK4*, and *GPR125* showed highly consistent expression in Cluster 3 of the three NB cohorts, with *CDK4* and *GPR125* predicting an AUC of greater than 0.7 for Cluster 1&2 and Cluster 3 in all three NB cohorts, implying good discriminatory ability (Figure 4F, 4G). Interestingly, in combination with clinical information, we found that *CDK4* performed best in predicting the prognosis of patients in three NB cohorts, which is an essential tumor driver gene and may be a crucial prognostic marker for the Cluster 3 (Figure 4H) [54–56]. In conclusion, by analyzing multiple perspectives, we found that *GREB1*, *CDK4*, and *GPR125* may be the critical markers of Cluster 3 in NB, especially *CDK4* and *GPR125*. Clinically, by suppressing the expression of these genes, it may have important to improve prognosis for NB patients.

### Drug sensitivity assessment for Fges-derived subtypes

**Figure 5:**
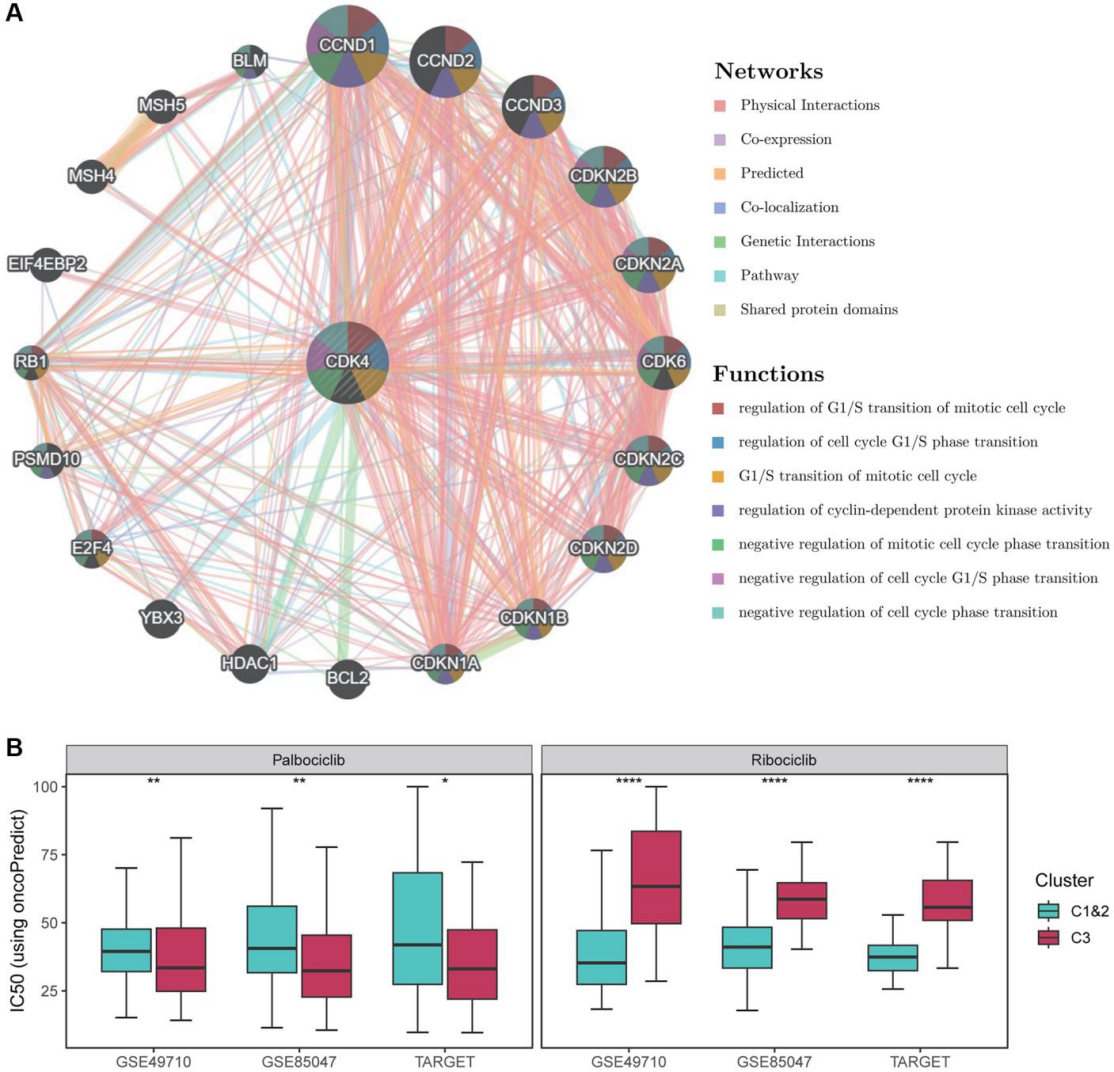
**Drug sensitivity analysis of *CDK4* inhibitors.** (**A**) Functional analysis of the protein-protein interaction network and *CDK4*, along with its neighboring genes. The edges of the network are color-coded to indicate the bioinformatics methods used, including physical interactions, co-expression, site prediction, co-localization, pathway and genetic interactions, and shared protein structural domains. The nodes of the network are also color-coded to reflect the enrichment results of the genome. The size of each circle corresponds to the rank of the gene associated with *CDK4*, while the width of each line represents the weight of the data source used in the composite network. (**B**) Box plots show the distribution of IC50 values for Palbociclib as well as Ribociclib in Fges-derived subtypes. *P*-values were obtained from *t*-tests. ^*^p < 0.05; ^**^p < 0.01; ^****^p < 0.0001.

Considering the potential clinical application of *CDK4* in Fges-derived Cluster 3, we used GeneMANIA to construct a protein-protein interaction network and identify genes associated with *CDK4*. The results showed that *CDK4* is involved in cell cycle as well as regulatory activities (Figure 5A). This finding further suggests that high expression of this gene promotes the proliferation of tumor cells. Currently, *CDK4*-targeted drug inhibitors are an emerging approach to cancer treatment, with several drugs already approved by the Food and Drug Administration (FDA) and showing good efficacy in clinical practice. For example, Palbociclib [57, 58] can halt tumor cell proliferation by inhibiting the G1/S phase transition of the cell cycle, while Ribociclib inhibitors prevent the binding of *CDK4*/*6* to D-type cyclin, thereby blocking the G1/S phase transition [59–61].

To determine the sensitivity of NB subtypes Cluster 1&2 and Cluster 3 to Palbociclib and Ribociclib inhibitors, we used OncoPredict tool [47] to predict the distribution of IC50 values for NB patients. Our results showed that the IC50 values of Palbociclib were consistently lower and significantly different in Cluster 3 compared to Cluster 1&2 (Figure 5B; see Materials and Methods). In contrast, Ribociclib had consistently higher IC50 values in Cluster 1&2 than in Cluster 3 (Figure 5B). Therefore, Palbociclib could be a potentially promising drug target for the treatment of Cluster 3, while Ribociclib may have an important therapeutic role for Clusters 1&2.

### Expression suppression of MHC class II in myeloid cells drives poor prognosis in Cluster 3 subtype of NB

**Figure 6:**
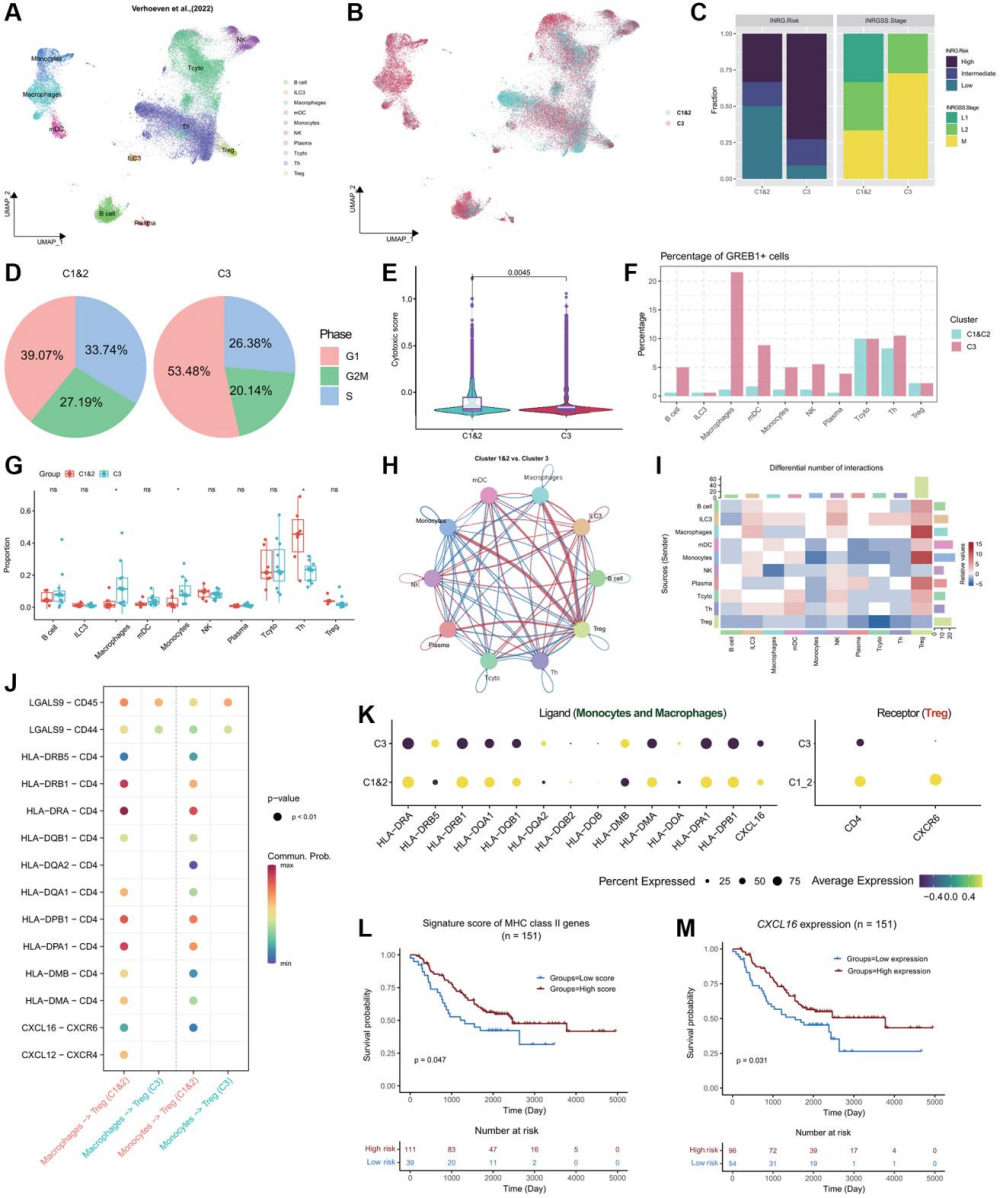
**Single-cell profiling of the NB immune microenvironment and subtype-specific interactions.** (**A**) Global overview of NB immune cell atlas containing 46,134 cells, color coded by annotated cell type (*n* = 17). (**B**) Global overview of NB immune cell atlas, color coded by predicted NB subtypes. (**C**) Stacked bar plots showing the distribution of Cluster 1&2 and Cluster 3 subtypes of NB patients in the INRG (left panel) and INRGSS (right panel) classifications. (**D**) Pie plots showing the percentage of cells in different NB subtypes in the G1, G2M, and S phases. (**E**) Violin plots combined with boxplots show the distribution of cytotoxic scores of cells in Cluster 1&2 and Cluster 3 subtypes. *P*-value was obtained from *t*-test. (**F**) Grouped bar plot showing the percentage of *GREB1*+ cells in different immune cell types of Cluster 1&2 and Cluster 3 patients. (**G**) Grouped boxplot showing the cellular proportion of Cluster 1&2 and Cluster 3 patients. *P*-values were obtained from *t*-tests. ns, not significant; ^*^p < 0.05. (**H**) Interaction map depicting the ligand-receptor interactions within the NB immune microenvironment. Red indicates stronger interactions between ligands and receptors in Cluster 1&2 compared to Cluster 3, while blue indicates weaker interactions in Cluster 1&2 compared to Cluster 3. The thickness of the lines represents the strength of the differences. (**I**) Heatmap showing the differences in ligand-receptor interactions between Cluster 1&2 and Cluster 3. Red indicates stronger interactions between ligands and receptors in Cluster 1&2 compared to Cluster 3, while blue indicates weaker interactions in Cluster 1&2 compared to Cluster 3. (**J**) Bubble plot showing the significant interactions between receptor and ligand genes among Monocytes, Macrophages, and Treg cells in both Cluster 1&2 and Cluster 3 subtypes (*p* < 0.01). The color gradient ranging towards red indicates stronger interactions. (**K**) Bubble plot showing the expression of ligands MHC II molecules and *CXCL16* in Monocytes and Macrophages of both Cluster 1&2 and Cluster 3 subtypes, as well as the expression of receptor genes *CD4* and *CXCR6* in Treg cells. (**L**) KM curve showing the stratification derived by the signature score of MHC Class II genes exhibiting significant differences in prognosis. *P*-value was obtained by log-rank test. (**M**) KM curve showing the stratification derived by the expression of *CXCL16* exhibiting significant differences in prognosis. *P*-value was obtained by log-rank test.

Based on the study conducted by Verhoeven et al. (2022) [5], we obtained a comprehensive single-cell dataset comprising 46,134 cells from 17 neuroblastoma (NB) donors (Figure 6A; Supplementary Table 9). This dataset encompasses 10 crucial immune cell types, including B cells, ILC3, Macrophages, mDC, Monocytes, NK cells, Plasma cells, Tcyto, Th, and Treg cells. Employing a pre-trained XGBoost model, we successfully predicted the subtypes of the 17 patients, namely Cluster 1&2 and Cluster 3. Out of these patients, 6 were classified as C1&2 subtype, while the remaining 11 were identified as Cluster 3 subtype (Figure 6B; Supplementary Table 9). By integrating the clinical information associated with these NB patients, we observed a distinct pattern among the subtypes. Cluster 3 subtype patients were predominantly situated in the high-risk INRG category and exhibited the INRGSS M stage (Figure 6C). In contrast, Cluster 1&2 subtype NB patients displayed a higher prevalence in the low-risk INRG category and were predominantly in the INRGSS L1 and L2 stages (Figure 6C). To investigate the molecular characteristics specific to the C1&2 and C3 subtypes in the NB patients, we scrutinized the cell cycle distribution. Notably, Cluster 3 subtype cells exhibited a pronounced accumulation in the G1 phase (53.48%), indicating an elevated proliferative capacity (Figure 6D). Additionally, the cytotoxicity scores of Cluster 1&2 subtype were significantly higher compared to those of Cluster 3, aligning with the features observed in the NB-TARGET bulk classification (Figures 3C and 6E). In terms of gene expression, *GREB1* emerged as the important distinctive gene feature in the C3 subtype, with the highest proportion of positive expression observed in Macrophages (Figure 6F). Abundance analysis across different cell types within the Cluster 1&2 and Cluster 3 subtypes demonstrated a substantial enrichment of Macrophages and Monocytes in Cluster 3, while Th cells exhibited a significantly higher abundance in Cluster 1&2 (Figure 6G).

To investigate the variations in cell-to-cell signaling and interactions between the Cluster 1&2 and Cluster 3 subtypes and gain insights into the unfavorable prognosis associated with the Cluster 3 subtype, we employed CellChat [48] for cell-cell interaction analysis. Our findings revealed significantly stronger interactions between Macrophages, Monocytes, and Treg cells in the Cluster 1&2 subtype compared to the Cluster 3 subtype (Figure 6H, 6I). Further analysis of receptor and ligand genes highlighted a notable deficiency in the interaction between MHC Class II molecules and *CD4* in Cluster 3, as well as the *CXCL16*-*CXCR6* axis interaction (Figure 6J). Additionally, Monocytes and Macrophages within the Cluster 3 subtype exhibited suppressed expression of MHC Class II genes and *CXCL16*, indicating an unfavorable prognosis for NB patients (Figure 6K–6M). In summary, our analysis emphasizes the downregulation of MHC Class II and *CXCL16* expression in Monocytes and Macrophages within the Cluster 3 subtype, leading to weakened interactions with Treg cells and ultimately contributing to a poorer prognosis for patients with the Cluster 3 subtype.

## DISCUSSION

NB is a childhood cancer that develops from immature nerve cells in the sympathetic nervous system. However, current clinical and molecular subtyping methods for NB have limitations, and may not always provide accurate prognostic information or guide treatment decisions [12, 62, 63]. This leads to challenges in predicting clinical outcomes and selecting appropriate treatments, highlighting the need for new approaches to better classify NB and improve disease management. To address this challenge, we explored the microenvironment of NB using Fges, which revealed some degree of heterogeneity that could potentially indicate subtypes. This speculation was supported when we performed consensus clustering of the Fges activity scores. Three subtypes (Cluster 1, Cluster 2, and Cluster 3) derived by Fges demonstrated significant differences in prognosis compared to the current mainstream NB subtypes (i.e., COG, and INSS) [9], providing a new strategy for NB tying.

By assessing the degree of immune infiltration, immunogenicity, CD8T cytotoxicity, and tumor purity of the Fges-derived three subtypes, we were able to shed light on their respective biological functions. Our analysis showed that Cluster 1 and Cluster 2 were more immunoreactive, while Cluster 3 demonstrated significantly higher tumor purity, which is consistent with its poor prognosis. Furthermore, gene ontology annotation and pathway analysis revealed that the three subtypes are associated with distinct biological processes. Specifically, Cluster 1 is involved in immune activation, Cluster 2 is strongly associated with EMT, while Cluster 3 is enriched in biological processes or pathways such as cell cycle processes. It is important to note that previous studies [64–66] have shown a correlation between EMT activity and the ability of NB tumors to infiltrate and metastasize, ultimately, leading to worse prognosis. However, it is important to recognize that NB is a complex tumor and that different subtypes of NB may exhibit distinct biological features and clinical manifestations. Therefore, the prognostic impact of EMT activity may differ for different subtypes of NB. Additionally, further studies are necessary to investigate the role of EMT in the development and progression of NB.

To better extend Fegs-derived subtypes in other NB cohorts, we developed a classification model using XGBoost to predict Cluster 1&2 versus Cluster 3. After evaluation, the model exhibited good performance in the training set (accuracy = 0.95). In the independent NB cohorts, the subtypes predicted by the classifier showed significant prognostic differences consistent with the expected pattern, which is crucial for investigating the Fges-derived subtypes in more extensive NB cohorts. Specifically, we found *GPR125*, *CDK4*, and *GREB1* to be the most significant marker genes in the Cluster 3 subtype. Our study suggests that these genes may play a crucial role in NB and that their overexpression or aberrant activation is closely associated with tumorigenesis, progression, and metastasis [54, 56, 66], and may be viable therapeutic targets for NB. Furthermore, our findings potentially shed light on the underlying reasons for the poor prognosis of Cluster 3. Given the significant impact of the *CD4K* gene on prognosis, we conducted further research on the drug guidance of *CD4K* inhibitors on Fges-derived subtypes, and found that Palbociclib was more sensitive in the Cluster 3, while Ribociclib performed well in the Cluster 1&2. This suggests that subtyping of NB may help in selecting appropriate treatment strategies to achieve better therapeutic effects.

In a separate single-cell analysis conducted on NB patients, we examined the characteristics and interactions among different subtypes. The study unveiled unfavorable clinical prognostic features associated with the Cluster 3 subtype, including high-risk classification, increased proliferative capacity, and lower cytotoxicity scores. Moreover, compared to Cluster 1&2, the expression of MHC-II and *CXCL16* in monocytes and macrophages within Cluster 3 was suppressed, leading to weakened interactions with Treg cells, thus influencing patient prognosis. Specifically, Tregs, known for their immune regulatory role, typically interact with monocytes to maintain immune balance. However, the diminished interaction between monocytes presenting MHC class II molecules and *CD4* molecules in Tregs resulted in reduced suppressive effects by Tregs, leading to weakened immune regulation. Consequently, this could potentially trigger excessive or imbalanced immune responses, causing self-tissue damage or triggering a stronger immune response against tumor antigens. This could potentially result in excessive or imbalanced immune responses, causing damage to self-tissues or generating a stronger immune response against tumor antigens [67, 68]. These findings contribute significantly to our understanding of the differences between NB subtypes and the interactions among immune cells, offering valuable insights for the development of more effective treatment strategies.

Despite the promising results, our study has some limitations. Firstly, the study was based on a relatively small sample size, and more extensive validation is needed. Secondly, we only investigated two drugs, and more drugs need to be examined in future studies. Thirdly, further investigation is needed to validate the clinical utility of our subtyping approach. Nonetheless, our study highlights the potential of using Fges to derive novel subtypes of NB with significant differences in prognosis and treatment response. In conclusion, as research in NB subtyping and treatment strategies continues to advance, a multidisciplinary approach involving clinicians, researchers, and patients will be pivotal in translating these findings into more effective, personalized, and less burdensome treatment options. The ultimate goal is to enhance patient outcomes and the overall experience of those affected by NB.

## Supplementary Materials

Supplementary Tables 4, 8 and 9

Supplementary Table 1

Supplementary Table 2

Supplementary Table 3

Supplementary Table 5

Supplementary Table 6

Supplementary Table 7
