# Supplementary material for: Immune microenvironment heterogeneity reveals distinct subtypes in neuroblastoma: insights into prognosis and therapeutic targets
Source: Aging (Albany NY). 2023 Nov 27;15(22):13345–67. doi: 10.18632/aging.205246 (PMC10713432; doi:10.18632/aging.205246)
Supplement: Supplementary Tables 4, 8 and 9 [file aging-15-205246-s001.pdf]

SUPPLEMENTARY TABLES

Supplementary Table 4. Prognostic effect of Fges activity scores in TARGET-NB cohort.

	HR	*P*-value
MHCI	1.821275179	0.039615164
MHCII	0.583836443	0.03041356
Coactivation_molecules	1.860932833	0.043129543
Effector_cells	0.636452651	0.13882156
T_cell_traffic	0.69708204	0.122197637
NK_cells	0.571949912	0.030198616
T_cells	0.634409842	0.078006928
B_cells	2.391219091	0.090508321
M1_signatures	0.723246517	0.163329204
Th1_signature	0.701370599	0.135638806
Antitumor_cytokines	0.50783405	0.032018353
Checkpoint_inhibition	0.51236251	0.024542153
Treg	0.489392939	0.002301173
T_reg_traffic	0.461730268	0.00401174
Neutrophil_signature	0.558161447	0.064353074
Granulocyte_traffic	0.664911796	0.085467705
MDSC	0.539404379	0.01864849
MDSC_traffic	0.556755849	0.028336472
Macrophages	0.712436722	0.149556066
Macrophage_DC_traffic	0.646581481	0.066628528
Th2_signature	0.60954905	0.086528464
Protumor_cytokines	1.761820619	0.015157943
CAF	1.674823647	0.06215062
Matrix	0.66364449	0.100221482
Matrix_remodeling	2.429286849	0.009150648
Angiogenesis	2.089288111	0.111961105
Endothelium	0.793468888	0.369515344
Proliferation_rate	2.803623103	0.000405439
EMT_signature	2.229806924	0.083548746

Supplementary Table 8. **List of features used for training XGBoost model.**

hgnc_symbol	ensembl_gene_id	chromosome_name	start_position	end_position	description
GPR125	ENSG00000152990	4	22345071	22516066	adhesion G protein-coupled receptor A3 [Source:HGNC Symbol;Acc:HGNC:13839]
AHR	ENSG00000106546	7	16916359	17346152	aryl hydrocarbon receptor [Source:HGNC Symbol;Acc:HGNC:348]
CCL13	ENSG00000181374	17	34356480	34358610	C-C motif chemokine ligand 13 [Source:HGNC Symbol;Acc:HGNC:10611]
CCNB2	ENSG00000157456	15	59105126	59125045	cyclin B2 [Source:HGNC Symbol;Acc:HGNC:1580]
CD2	ENSG00000116824	1	116754430	116769229	CD2 molecule [Source:HGNC Symbol;Acc:HGNC:1639]
CD22	ENSG00000012124	19	35319261	35347361	CD22 molecule [Source:HGNC Symbol;Acc:HGNC:1643]
CD3E	ENSG00000198851	11	118304730	118316175	CD3 epsilon subunit of T-cell receptor complex [Source:HGNC Symbol;Acc:HGNC:1674]
CD52	ENSG00000169442	1	26317958	26320523	CD52 molecule [Source:HGNC Symbol;Acc:HGNC:1804]
CD79A	ENSG00000105369	19	41877279	41881372	CD79a molecule [Source:HGNC Symbol;Acc:HGNC:1698]
CDK4	ENSG00000135446	12	57747727	57756013	cyclin dependent kinase 4 [Source:HGNC Symbol;Acc:HGNC:1773]
CEBPD	ENSG00000221869	8	47736913	47738164	CCAAT enhancer binding protein delta [Source:HGNC Symbol;Acc:HGNC:1835]
CENPF	ENSG00000117724	1	214603185	214664574	centromere protein F [Source:HGNC Symbol;Acc:HGNC:1857]
CKM	ENSG00000104879	19	45306413	45322875	creatine kinase, M-type [Source:HGNC Symbol;Acc:HGNC:1994]
COL11A1	ENSG00000060718	1	102876467	103108872	collagen type XI alpha 1 chain [Source:HGNC Symbol;Acc:HGNC:2186]
CPNE7	ENSG00000178773	16	89575758	89597246	copine 7 [Source:HGNC Symbol;Acc:HGNC:2320]
CXCL10	ENSG00000169245	4	76021118	76023497	C-X-C motif chemokine ligand 10 [Source:HGNC Symbol;Acc:HGNC:10637]
FCER2	ENSG00000104921	19	7688758	7702146	Fc epsilon receptor II [Source:HGNC Symbol;Acc:HGNC:3612]
FAIM3	ENSG00000162894	1	206903317	206923247	Fc mu receptor [Source:HGNC Symbol;Acc:HGNC:14315]
FN1	ENSG00000115414	2	215360440	215436073	fibronectin 1 [Source:HGNC Symbol;Acc:HGNC:3778]
FOSB	ENSG00000125740	19	45467995	45475179	FosB proto-oncogene, AP-1 transcription factor subunit [Source:HGNC Symbol;Acc:HGNC:3797]
FOXD3	ENSG00000187140	1	63322567	63325128	forkhead box D3 [Source:HGNC Symbol;Acc:HGNC:3804]
GADD45B	ENSG00000099860	19	2476122	2478259	growth arrest and DNA damage inducible beta [Source:HGNC Symbol;Acc:HGNC:4096]
GREB1	ENSG00000196208	2	11482341	11642788	growth regulating estrogen receptor binding 1 [Source:HGNC Symbol;Acc:HGNC:24885]
HMOX1	ENSG00000100292	22	35380361	35394214	heme oxygenase 1 [Source:HGNC Symbol;Acc:HGNC:5013]
HP	ENSG00000257017	16	72054505	72061055	haptoglobin [Source:HGNC Symbol;Acc:HGNC:5141]
IGFBP1	ENSG00000146678	7	45888360	45893660	insulin like growth factor binding protein 1 (Source:HGNC Symbol;Acc:HGNC:5469
ITGA8	ENSG00000077943	10	15513954	15719922	integrin subunit alpha 8 [Source:HGNC Symbol;Acc:HGNC:6144]

KLF4	ENSG00000136826	9	107484852	107490482	KLF transcription factor 4 [Source:HGNC Symbol;Acc:HGNC:6348]
LCK	ENSG00000182866	1	32251244	32286165	LCK proto-oncogene, Src family tyrosine kinase [Source:HGNC Symbol;Acc:HGNC:6524]
LGI4	ENSG00000153902	19	35124513	35142451	leucine rich repeat LGI family member 4 [Source:HGNC Symbol;Acc:HGNC:18712]
FAM60A	ENSG00000139146	12	31280584	31327058	SIN3-HDAC complex associated factor [Source:HGNC Symbol;Acc:HGNC:30702]
C8orf4	ENSG00000176907	8	40153482	40155310	transcriptional and immune response regulator [Source:HGNC Symbol;Acc:HGNC:1357]

Supplementary Table 9. Clinical and subtyping information of 19 NB single-cell datasets.

Sample. ID	Age. at. sampling	Risk. group. INRG. at. diagnosis	Stage. INRGSS.	Predict
NB01	4	Low	L1	C1&2
NB02	18	Intermediate	L2	C3
NB09	82	High	M	C3
NB11	138	Intermediate	L2	C1&2
NB12	101	High	M	C3
NB13	48	High	M	C3
NB15	76	High	M	C1&2
NB16	23	High	L2	C3
NB17	63	High	M	C1&2
NB18	111	High	M	C3
NB19	6	Low	L2	C3
NB20	83	High	M	C3
NB23	7	Intermediate	M	C3
NB24	128	High	M	C3
NB26	4	Low	L1	C1&2
NB34	100	High	M	C3
NB37	60	Low	L2	C1&2
